# Family Identification and Functional Study of Copper Transporter Genes in *Pleurotus ostreatus*

**DOI:** 10.3390/ijms252212154

**Published:** 2024-11-12

**Authors:** Lifeng Guo, Tonglou Li, Baosheng Zhang, Kexing Yan, Junlong Meng, Mingchang Chang, Ludan Hou

**Affiliations:** 1 College of Food Science and Engineering, Shanxi Agricultural University, Jinzhong 030801, China; m17836509709@163.com (L.G.); 15582690226@163.com (T.L.); 15291884191@163.com (B.Z.); yan15934475351@163.com (K.Y.); mengjunlongseth@hotmail.com (J.M.); 2 Shanxi Research Center for Engineering Technology of Edible Fungi, Jinzhong 030801, China; 3 Shanxi Key Laboratory of Edible Fungi for Loess Plateau, Jinzhong 030801, China

**Keywords:** *Pleurotus ostreatus*, copper transporters, cell membrane, overexpression, RNA interference, heat stress

## Abstract

The copper transport (COPT/Ctr) family plays an important role in maintaining metal homeostasis in organisms, and many species rely on Ctrs to achieve transmembrane transport via copper (Cu) uptake. At present, the Ctr family is widely studied in plants. However, there are few reports on the use of Ctrs in edible mushrooms. In this study, the *Pleurotus ostreatus* CCMSSC00389 strain was used as the research object, and the addition of exogenous copper ions (Cu^2+^) increased the temperature tolerance of mycelia, maintained the integrity of cell membranes, and increased mycelial density. In addition, four *PoCtr* genes were further identified and subjected to bioinformatics analysis. Further research revealed that there were differences in the expression patterns of the *PoCtr* genes under different temperature stresses. In addition, the biological function of *PoCtr4* was further explored by constructing transformed strains. The results showed that OE-*PoCtr4* enhanced the tolerance of mycelia to heat stress and H_2_O_2_. After applying heat stress (40 °C), OE-*PoCtr4* promoted the recovery of mycelia. Under mild stress (32 °C), OE-*PoCtr4* promoted mycelial growth, maintained cell membrane integrity, and reduced the degree of cell membrane damage caused by heat stress. It is speculated that OE-*PoCtr4* may maintain the integrity of the cell membrane and enhance the heat resistance of mycelia by regulating the homeostasis of Cu^2+^.

## 1. Introduction

*Pleurotus ostreatus* is among the primary varieties of edible mushrooms found in China [1]. However, in China, *P. ostreatus* is mainly cultivated via agricultural methods, with poor equipment and facilities and a poor environmental regulation ability, so it often faces environmental stresses such as high or low temperatures during cultivation. Moreover, a high temperature is the most unfavorable stress in the culturing of *P. ostreatus*, because the growth of mycelia is not only inhibited after high-temperature stress, but also easily contaminated by mold [2]. These unfavorable factors cause great losses to mushroom farmers every year, so the breeding of heat-tolerant strains has become a popular research topic in recent years.

Copper (Cu) is a vital trace element necessary for the survival of living organisms [3], and almost all cell types require Cu for various physiological processes [4]. Copper ions (Cu^+^) are important enzyme affinity groups, and Cu homeostasis is strictly regulated [5]. Moderate amounts of the trace element Cu can help plants to cope with various abiotic and biotic stresses [6]. However, in plants, Cu deficiency can lead to a decrease in growth rate and result in different phenotypes, such as curled leaf margins, the yellowing of young leaves, impaired fruit and seed formation, developmental defects, and reduced survival rates of seeds and pollen [7]. High levels of Cu are harmful because of the ability of free Cu^2+^ ions to generate reactive oxygen species, including superoxide and hydrogen peroxide, which can damage DNA, proteins, and lipids, ultimately damaging cells [8]. Adding micromolar concentrations of Cu^2+^ to the culture medium can significantly increase the growth of the brewing yeast *Saccharomyces cerevisiae* [9]. The production of melanin may be associated with the stimulating influence of Cu on laccases. In the presence of Cu, the white rot fungus *Trametes multicolor* enhances laccase activity [10]. However, the function of Cu in the growth and development of edible mushrooms remains ambiguous.

Copper transporters (Ctrs) play crucial roles in maintaining Cu homeostasis [11] and participate in the regulation of Cu^+^ homeostasis [12], including absorption [13], transport [14], utilization [15], storage [16], and clearance [17]. Research has shown that three Ctrs (RiCtr1-3) are involved in fungal *Rhizophagus* irregularity. Among them, RiCtr1 mediates the absorption of Cu from the environment by extraradical mycelia, RiCtr2 participates in the mobilization of Cu vacuole storage, and RiCtr3a participates in copper tolerance [18]. In addition, it plays an important role in regulating the effects of Cu^+^ on oxidative stress, energy production, melanin formation, neuropeptide biosynthesis, and connective tissue maturation [19]. The earliest research on Cu homeostasis can be traced back to the cloning of the Cu metallothionein gene CUP1 and the transcription factor ACE1, which regulate its expression in brewing yeast [20]. Recently, Ctrs have been extensively studied in fields such as plants. For example, in *Arabidopsis thaliana*, the knockout of Ctrs affects the absorption of Cu^+^ and pollen development [21]. In Cu-tolerant rice varieties, the increase in Ctrs is significantly greater than that in Cu-sensitive rice varieties [22]. In apples, excess Cu can be stored in the cell walls of apple roots and stems via Ctrs to increase Cu tolerance [23]. Researchers have identified four distinct types of Ctrs in *Aspergillus fumigatus*, with *ctrA2* and *ctrC* cloned from *A. fumigatus*, and it was found that they can functionally supplement *Ctr1* in *S.cerevisiae* [24]. In *Lycium barbarum* mycorrhizal roots, Ctrs can improve the growth and nutrient absorption of tobacco [25]. In addition, studies have shown that the Ctrs on the surface of fungal cells are one of the necessary conditions for completing the spore germination program [26]. However, to date, the function of the *Ctr* gene in edible mushrooms has not been studied.

In this study, the addition of trace amounts of exogenous Cu^2+^ increased the resistance of mycelia. In addition, four *PoCtrs* were identified and subjected to bioinformatics analysis. The biological function of *PoCtr4* was subsequently explored by constructing transformed strains. The aim of this study was to lay the foundation for a further exploration of how Ctrs regulate the growth and development mechanisms of *P. ostreatus*.

## 2. Results

### 2.1. Different Heat Stress Treatments Affect the Integrity of the Mycelial Cell Membrane of P. ostreatus

**Figure 1:**
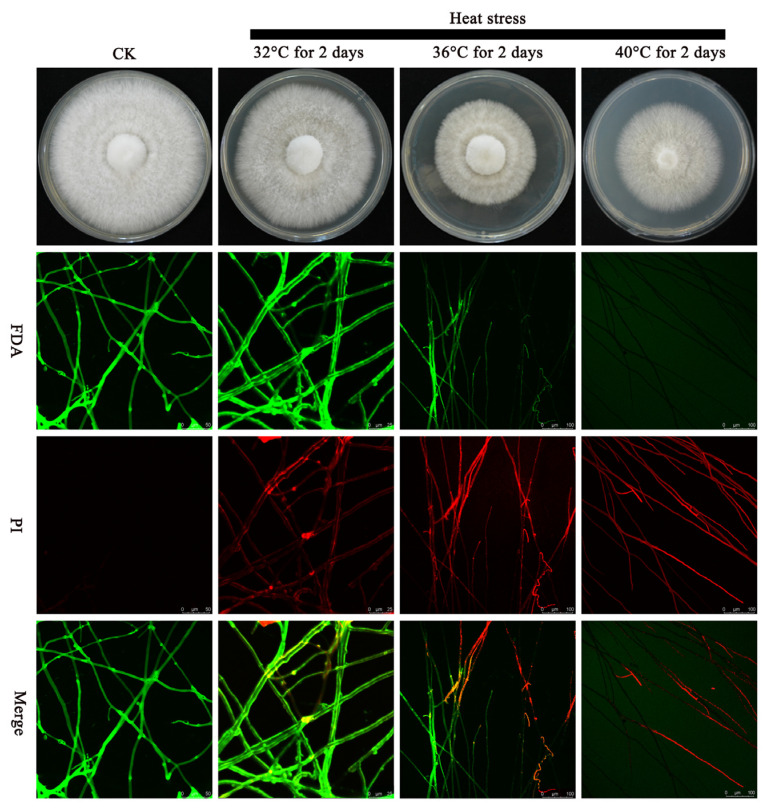
Effects of different temperature stresses on the integrity of the cell membrane of *P. ostreatus* mycelia on PDA plates. (Control Check: CK; Fluorescein diacetate: FDA; Propidium iodide: PI; potato dextrose agar: PDA).

There are significant differences in the inhibitory effects of different heat stresses on the growth rate of edible mushroom mycelia. Research has indicated that heat stress can cause ROS explosions within mycelia, affecting cell membrane integrity and leading to cell damage [27]. At present, propidium iodide (PI) and fluorescein diacetate (FDA) fluorescent probes have been widely used in the detection of cell viability. Research has shown that when the membrane is intact, PI enters the cell, but is released with the help of transporters. On the other hand, if the membrane is compromised, the dye enters, but is not removed, resulting in the staining of damaged cells. At this time, FDA mainly binds to intracellular lipase to produce fluorescein, thereby producing a green fluorescence signal. When the integrity of the cell membrane is compromised, PI penetrates the cell and associates with nucleic acid components (DNA and RNA) located within the cell, causing the fluorescence color to turn red and resulting in red fluorescence. Therefore, this study further investigated the effects of different heat stress treatments on the mycelia of *P. ostreatus* by using two fluorescent probes. As shown in Figure 1, when the stress temperature was 32 °C, the growth rate of mycelia was affected, the density of mycelia decreased, and red fluorescence appeared, whereas green fluorescence decreased compared with that of the control. When the stress temperature was 36 °C, the growth of mycelia was almost completely inhibited, and only a few fuzzy mycelia could be observed. Compared with that at 32 °C, the red fluorescence increased significantly, and the green fluorescence decreased. When the stress temperature was 40 °C, the mycelia stopped growing, and only red fluorescence was observed.

In summary, as the temperature increased, the integrity of the cell membrane gradually decreased and the survival rate of the mycelial cells of *P. ostreatus* decreased.

### 2.2. The Addition of Exogenous Copper Ions Enhances the Tolerance of Mycelia to Heat Stress

**Figure 2:**
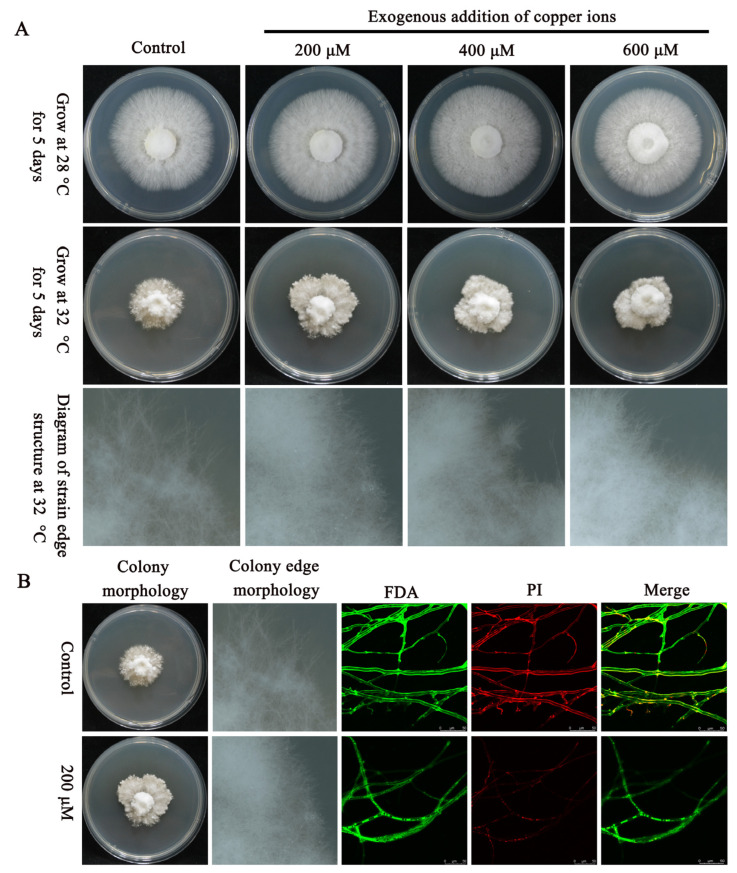
Effects of temperature and Cu^2+^ on *P. ostreatus* strains. (**A**) Effects of different Cu^2+^ concentrations at 28 °C and 32 °C on the phenotypes of *P. ostreatus* strains. (**B**) Apparent effects of 0 μM and 200 μM Cu^2+^ on the mycelia of *P. ostreatus* strains at 32 °C and changes in cell membrane integrity under laser confocal microscopy.

Cu^+^ is an essential cofactor for various important enzymes and can affect the activity of multiple enzymes. This study revealed that the addition of certain concentrations of exogenous Cu^2+^ (0 μM, 200 μM, 400 μM, or 600 μM) had no significant effect on the growth rate or colony density of *P. ostreatus* mycelia. However, as the concentration of exogenous Cu^2+^ increased, the growth rate of mycelia was significantly inhibited (Figure S1). Interestingly, under 32 °C heat stress, compared with the control, the addition of a certain concentration of exogenous Cu^2+^ (200 μM, 400 μM, or 600 μM) not only significantly promoted the mycelial growth rate, but also significantly increased the density of colony edges. When the concentration of exogenous Cu^2+^ was 200 μM, the mycelial growth rate was greater than that of the other groups (Figure 2A). Further research revealed that, under 32 °C heat stress, the addition of exogenous Cu^2+^ (200 μM) significantly reduced the red fluorescence produced by the mycelia compared with that in the control group (Figure 2B). These findings indicate that the addition of Cu^2+^ plays a positive role in maintaining cell membrane integrity under heat stress.

In summary, the addition of exogenous Cu^2+^ can enhance the tolerance of mycelia to heat stress, possibly by acting as an enzyme cofactor in detoxifying enzymes or as a cofactor in iron transport.

### 2.3. Chromosomal (Chr) Mapping and Gene Structure of the Ctr Family of P. ostreatus

**Figure 3:**
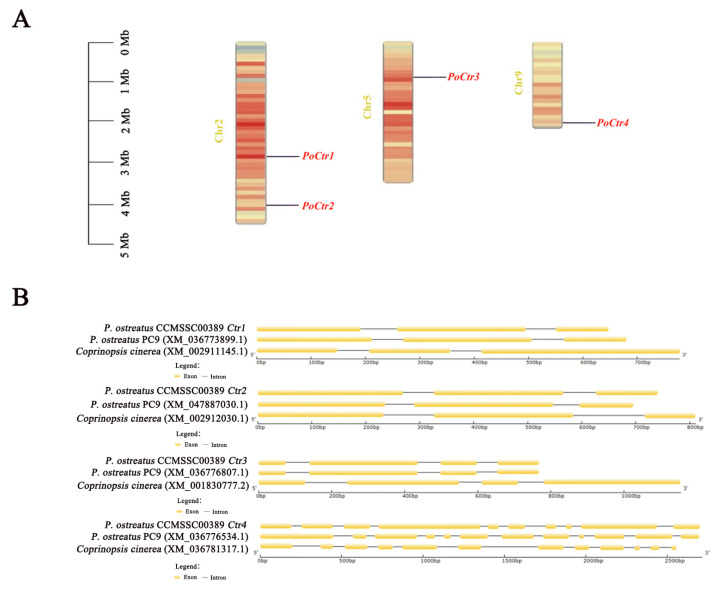
Chr mapping and gene structure of the fungus *PoCtrs*. (**A**) A schematic diagram of the distribution of the *Ctr* genes on the four Chrs of *P. ostreatus;* the gene names are shown in red on the right. The black line on the Chr indicates the location of the *PoCtr* genes. The left side of each Chr is where the Chr number can be found. (**B**) Gene structures of selected ctr-encoding genes in *P. ostreatus*, *Coprinopsis cinerea*, and *P. ostreatus* PC9. The exons are represented by yellow rectangles, and the black lines connecting two exons represent introns.

Ctr plays a crucial role in maintaining the homeostasis of Cu^+^ within cells. To investigate the relationship between genetic differentiation and gene replication within the *PoCtr* genes, four Ctr-encoding genes were identified from the genome of the *P. ostreatus* CCMSSC00389 strain and named *PoCtr1*, *PoCtr2*, *PoCtr3*, and *PoCtr4*. Afterwards, the positions of the *PoCtr* genes family on the Chr of *P. ostreatus* were determined. The results revealed that *PoCtr1* and *PoCtr2* were distributed on Chr2, that *PoCtr3* was distributed on Chr5, and that *PoCtr4* was distributed on Chr9 (Figure 3A). Second, the gene structure of the *PoCtr* genes family was further analyzed, and the results revealed that the full-length cDNA sequences of *PoCtr1*, *PoCtr2*, *PoCtr3*, and *PoCtr4* were 525, 624, 585, and 1980 bp long, respectively. DNA sequence analysis revealed that three exons in *PoCtr1* and *PoCtr2* were interrupted by two introns, four exons in *PoCtr3* were blocked by three introns, and ten exons in *PoCtr4* were interfered with by nine introns (Figure 3B).

### 2.4. Bioinformatics Analysis of PoCtr-Encoding Genes

To further investigate the function of the *PoCtrs* family in the growth and development of *P. ostreatus*, a detailed bioinformatics analysis was conducted first. The results revealed that PoCtr1 encodes a protein of 174 amino acids with a molecular weight of 19.06 kDa, a calculated isoelectric point of 9.60, an instability index (II) of 58.51, an aliphatic index of 101.67, and a hydrophilicity index of 0.361. PoCtr2 is a polypeptide consisting of 207 amino acid residues. Online prediction analyses indicated that the molecular weight of the PoCtr2 protein is approximately 22.53 kDa, with an isoelectric point of 8.48. The instability index (II) was calculated to be 31.36, indicating that the protein is stable. Additionally, the aliphatic index was recorded at 99.95, whereas the hydrophilicity index was 0.360. The online prediction analysis indicated that the molecular weight of the PoCtr3 protein is 21.00 kDa, the isoelectric point is 8.88, and the instability index (II) is 58.51, which classifies the protein as an unstable protein, with an aliphatic index of 101.67 and a hydrophilicity index of 0.361. PoCtr4 is a polypeptide chain consisting of 678 amino acid residues. Online prediction analyses indicated that the molecular weight of the PoCtr4 protein is approximately 73.95 kDa, with an isoelectric point of 6.21. The instability index (II) was calculated to be 33.32, which classified the protein as stable, with an aliphatic index of 91.03 and a hydrophilicity of 0.086 (Table 1).

**Figure 4:**
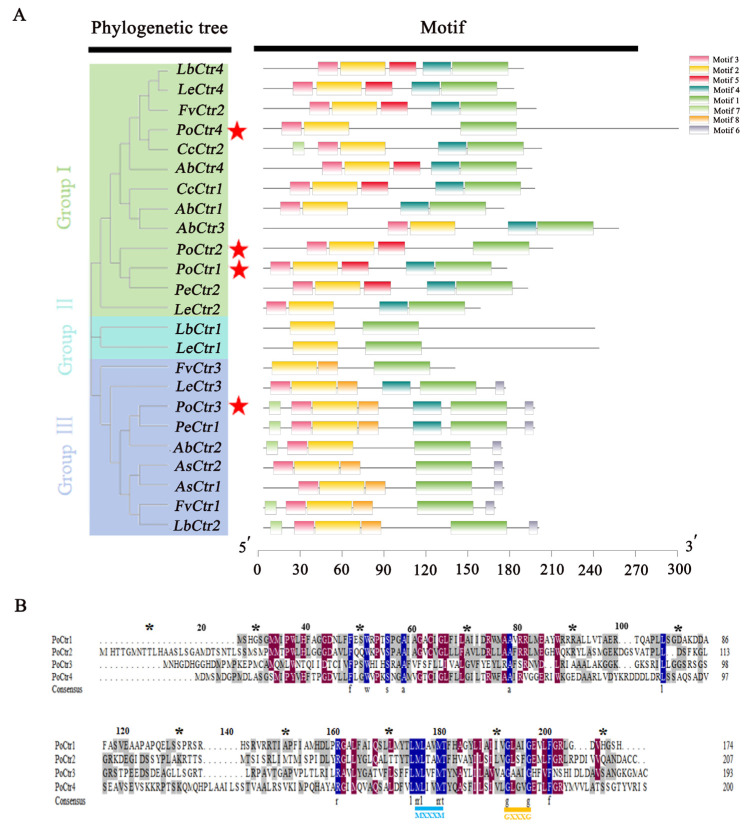
Amino acid alignment and conserved motif of PoCtrs. (**A**) Phylogenetic tree and motifs of the *P. ostreatus*, *Agaricus bisporus*, *Auricularia subglabra*, *C. cinerea*, *Flammulina velutipes*, *L. bicolor*, *Lentinula edodes*, and *P. eryngii* sequences. The red stars represent the four *PoCtr* genes of *P. ostreatus* in this study. (**B**) Amino acid sequences of four Ctrs identified from *P. ostreatus*; amino acid alignment was performed via MEGA7. The light blue and orange text show the (MxxxM and GxxxG) motifs in the expected transmembrane region, respectively, and the MxxxM and GxxxG motifs are closely related to Cu transporter function in the transmembrane structural region. The dark blue text represents 100%, the dark red text represents 75%, and the gray text represents 50% sequence identity and sequence similarity. The “*” above the sequence indicates every ten amino acid residues.

In addition, NCBI was used to predict the sequences of four PoCtr proteins, and the results revealed that they all contained conserved Ctr domains (Figure S2). Further conservative motif analyses revealed that motif 1 and motif 2 were present in all the PoCtr amino acid sequences. The two motifs in question may be associated with the PoCtr binding site. As illustrated in Figure 4A, four PoCtrs presented conserved motifs 1, 2, and 3. However, the motifs displayed separately for PoCtr1 were 4 and 5, the motif for PoCtr2 alone was 5, and the motifs for PoCtr3 alone were 4, 6, 7, and 8. The amino acid sequence alignment results revealed that the identities between PoCtr1, PoCtr1, PoCtr1, and PoCtr4 of *P. ostreatus* and the Ctrs of *Laccaria bicolor*, *C. cinerea*, and *Pleurotus eryngii* were 59.88%, 51.45%, 73.66%, and 56.08%, respectively (Figure S3). To clarify the homologous relationships among the four PoCtrs, amino acid comparisons among the four PoCtrs revealed that the homology of the four PoCtrs reached 38.47%, indicating that their relatives were distant. Most PoCtrs had an MxxxM motif within TMD2 and a GxxxG motif (Figure 4B). WoLF PSORT was employed to predict subcellular localization, and the four PoCtrs were all localized to the plasma membrane (Table 1).

### 2.5. Transmembrane Structure and 3D Structure Prediction of the PoCtrs Gene Family

**Figure 5:**
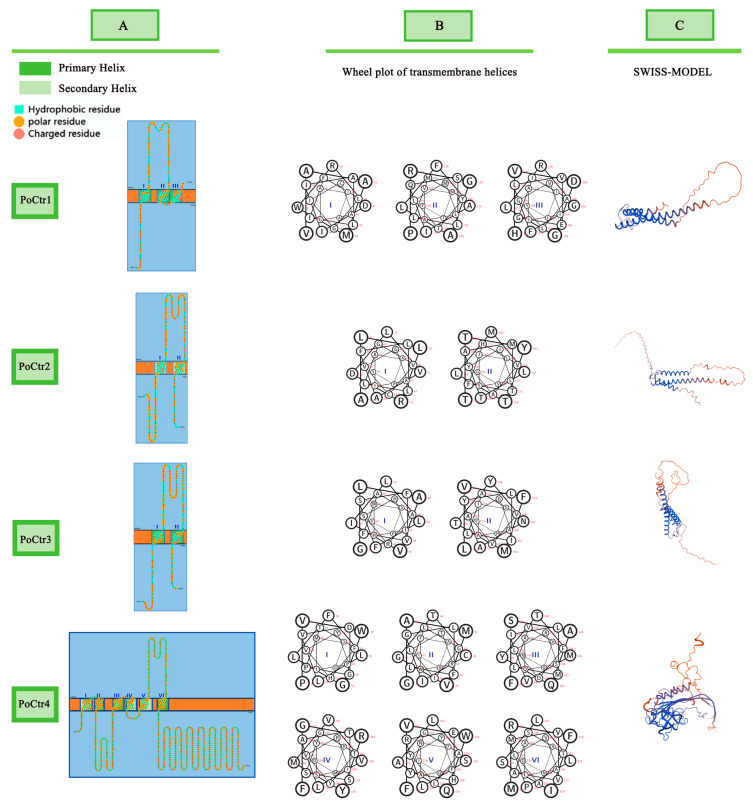
The PoCtrs transmembrane structures confirmed with the SOSUI tool (**A**) A wheel diagram of the transmembrane helix (**B**) and 3D structure homology models were predicted by SWISS-MODEL (**C**).

Three TMDs (TMDs 1, 2, and 3) are present in most Ctrs [28,29]. These “MxxxM motif” motifs are widely recognized as Mets (Cu binding or acquisition motifs—Mets). The MxxxM motif is essential for controlling transporter pore size and is required for the function of Ctrs, which is present in TMD2. The GxxxG motif is found in TMD3 and plays a crucial role in the close packing of the three transmembrane domains and the development of transporters that are both structurally and functionally mature. The transmembrane structure of PoCtrs can be achieved via the SOSUI tool, whereas its tertiary structure can be predicted via SWISS-MODEL (Figure 5A). The transmembrane structure revealed that PoCtr1 has three main transmembrane domains. PoCtr2 and PoCtr3 have two transmembrane domains, and PoCtr4 has three major transmembrane domains and three secondary transmembrane domains. A wheel diagram of the transmembrane helix is shown in Figure 5B. PoCtr4 has the most transmembrane spiral diagrams, with six diagrams. PoCtr1 has three transmembrane helix diagrams, and PoCtr2 and PoCtr3 have the fewest transmembrane helix diagrams, with only two each. Figure 5C shows the three-level structures of the four *PoCtrs*, which were predicted according to SWISS-MODEL.

### 2.6. Identification of Cis-Regulatory Elements in Four PoCtr Gene Promoters

**Figure 6:**
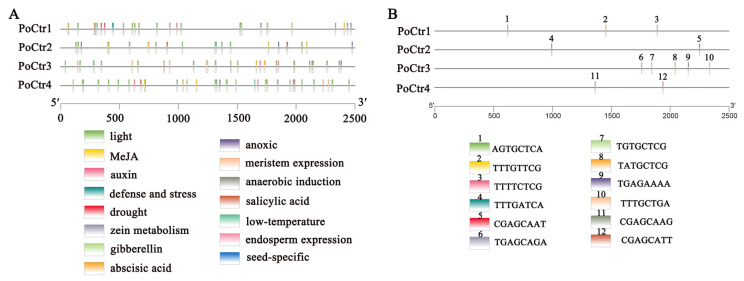
Regulatory elements known as common cis-regulatory elements (CREs) (**A**) and Cu-sensing transcription factor CREs (**B**) can be discovered in the *PoCtr* promoters. The vertical bars display the positional distribution of the projected CREs on the *PoCtr* promoters. PlantCARE was used to analyze the promoter sequences (2500 bp) of the four *PoCtr* genes. In this legend, each color represents different cis-elements.

After examining the 2500 bp upstream promoter sequence of the *PoCtr* genes, we investigated cis-elements, which may provide insights into the regulation of gene expression pathways. With respect to the Ctr gene family, the light-responsive gene has the most cis-elements (53). In addition, 59 cis-elements (i.e., MeJA (28), auxin (7), gibberellin (3), abscisic acid (19), and salicylic acid (2)) are involved in plant hormones. In the promoter sequence of the Ctr gene family, defense and stress, low temperature (3), drought (4), meristem expression (4), endosperm expression (1), zein metabolism (4), anoxic (6), and seed-specific (1) responses were also identified (Figure 6A). In addition, our previous research identified two copper-sensitive transcription factors (*PoMAC1a* and *PoMAC1b*) in the genome of *P. ostreatus* [30]. As shown in Figure 6B, the binding sites for Cu-sensitive transcription factors were further predicted in all four *PoCtr* promoter sequences. Changes in response components demonstrate the regulatory functions of the *PoCtrs* genes in many physiological and biological processes.

### 2.7. Collinearity Analysis of the PoCtrs Family

**Figure 7:**
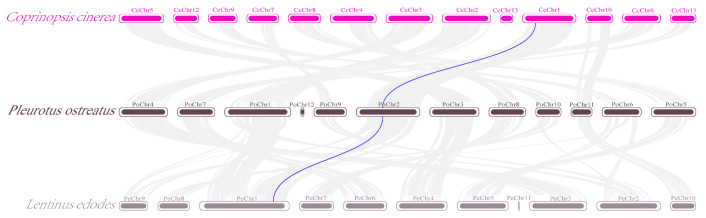
Homology analysis of the *Ctr* genes in the Chrs of *P. ostreatus*, *Lentinus edodes*, and *Coprinopsis cinerea* from model organisms. The gray line in the background highlights the homology of the *Ctr* gene pair, whereas the blue line highlights the colinear blocks in the genomes of *P. ostreatus* and two other fungi. The different colors of the boxes represent a variety of fungal varieties.

According to the collinearity analysis, *P. ostreatus*, *L. edodes*, and *C. cinerea* presented orthologs of the *Ctr* gene family (Figure 7). Briefly, on Chr 2, a *P. ostreatus* gene exhibited homology associations with an *L. edodes* gene and a *C. cinerea* gene. Notably, the homologs of *P. ostreatus* survived homology association with *C. cinerea* and *L. edodes*, suggesting that segmental duplication and genome-wide replication play significant roles in the evolutionary development of the *PoCtr* genes family.

### 2.8. Expression Patterns of the PoCtrs Gene Family

**Figure 8:**
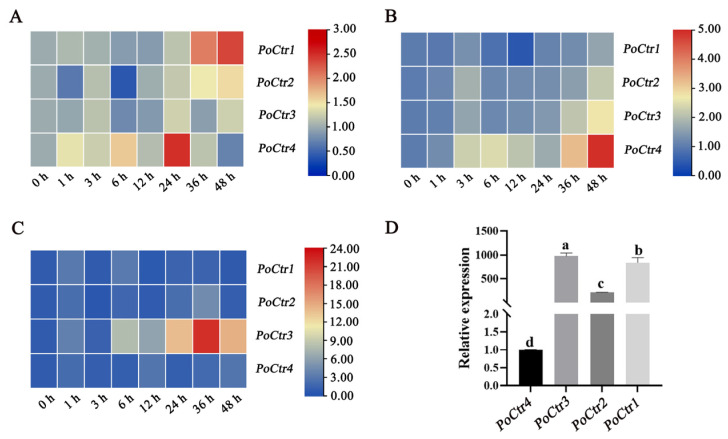
The expression pattern of the *PoCtr*-encoded genes at different temperatures at different times. (**A**) The expression pattern of *PoCtr* encoded genes under 32 °C stress for 2 days (d). (**B**) The expression pattern of *PoCtr*-encoded genes under 36 °C stress for 2 d. (**C**) The expression pattern of *PoCtr*-encoded genes under 40 °C stress for 2 d. (**D**) Determination of the relative expression of *PoCtr*-encoding genes (Different letters indicate significant differences for the comparison of samples (*p* < 0.05 according to Duncan’s test)).

To study the expression patterns of copper-transporter-encoding genes in response to thermal stress in *P. ostreatus*, the expressions of four different *Ctr* genes, *PoCtr1*, *PoCtr2*, *PoCtr3*, and *PoCtr4*, were analyzed under different durations of stress. The results of a Quantitative PCR (qPCR) experiment are shown in Figure 8.

Under 32 °C stress, the expression patterns of *PoCtr1* and *PoCtr2* gradually increased from 6 to 48 h (Figure 8A), and the phylogenetic tree revealed that *PoCtr1* and *PoCtr2* belonged to the same branch (Figure 4). It was inferred that *PoCtr1* and *PoCtr2* play major roles and perform similar functions at 32 °C. The relative expression level of *PoCtr3* did not significantly change at 32 °C. The expression pattern of *PoCtr4* first increased but then decreased at 32 °C, reaching its peak at 24 h (Figure 8A). At 36 °C, there was no significant trend in the expression pattern of *PoCtr1* (Figure 8B). The overall expression patterns of *PoCtr2*, *PoCtr3*, and *PoCtr4* tended to gradually increase, and their expression patterns were similar; however, the relative expression levels of *PoCtr4* were significantly upregulated by 4.96-fold at 48 h, and the relative expression levels of *PoCtr2* and *PoCtr3* were upregulated by 2.19- and 2.75-fold, respectively, after 48 h of heat stress (Figure 8B). It is inferred that *PoCtr4* plays the main role under 36 °C stress. At 40 °C, there was no significant trend in the expression patterns of *PoCtr1*, *PoCtr2* and *PoCtr4*. However, the expression pattern of *PoCtr3* first increased but then decreased, which suggested that *PoCtr3* may play a major function role at 40 °C (Figure 8C).

To gain deeper insights into the structure and function of *Ctr* genes in *P. ostreatus*, mycelia cultured at 28 °C were collected, and the expression levels of four *PoCtr*-encoding genes were compared and analyzed. The results indicated that the expression level of the *PoCtr4* gene was markedly lower than that of the other three encoding genes (Figure 8D). *PoCtr4* was located on a separate branch of the phylogenetic tree. Its special expression patterns at 32 °C and 36 °C and the unique minimum basal expression levels of the other three coding genes suggest that the *PoCtr4* gene may have more critical functions.

### 2.9. Acquisition of PoCtr4-Transformed Strains

**Figure 9:**
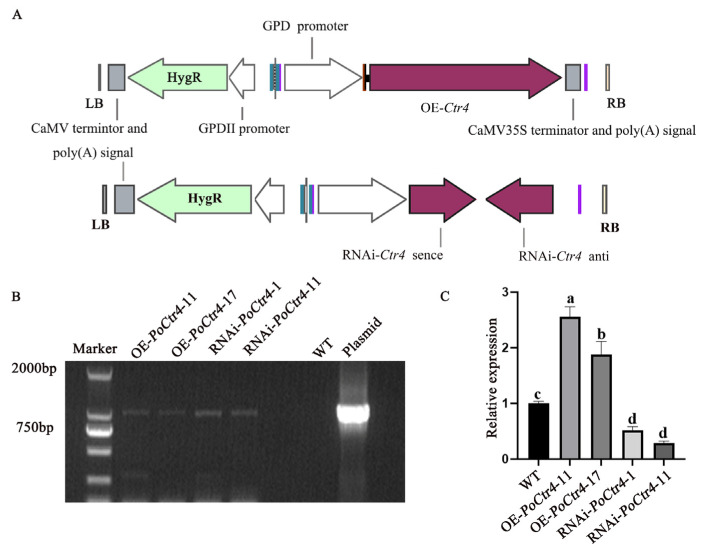
Screening and acquisition of *the PoCtr4*-transformed strains. (**A**) OE-*PoCtr4* and RNAi-*PoCtr4* plasmid maps. (**B**) Amplification of the *hyg* gene in *PoCtr4*-transformed strains. (**C**) Relative expression of *PoCtr4* in transformed strains (Different letters indicate significant differences in sample comparison, while the same letter indicates no significant differences in sample comparison (according to Duncan’s test, *p* < 0.05)).

In this study, the biological role of *PoCtr4* in *P. ostreatus* was investigated through the construction of transformed strains. Figure 9A shows the OE and RNAi plasmid maps of *PoCtr4*. Within the maps, the *hyg* gene served as a screening marker for the transformed strains. The OE-*PoCtr4* and RNAi-*PoCtr4* plasmids that were subsequently developed were subsequently introduced into the mycelia of *P. ostreatus* through *Agrobacterium tumefaciens*-mediated genetic transformation. First, the *hyg* gene fragment was amplified for further screening (Figure 9B). The expression of the *PoCtr4* gene in the recombinant strains was subsequently determined. Figure 9C shows that, compared with the WT strain, the expression of *Poctr4* was upregulated by 2.56-fold and 1.88-fold in the OE-*PoCtr*4-11 and OE-*PoCtr*4-17 strains, respectively. In contrast, in the RNAi-*PoCtr4*-1 and RNAi-*PoCtr4*-11 strains, the expression level of *PoCtr4* was downregulated by 48% and 72%, respectively. Therefore, these strains were selected for *PoCtr4* functional research.

### 2.10. PoCtr4 Positively Regulates the Response of Mycelia to Heat Stress

**Figure 10:**
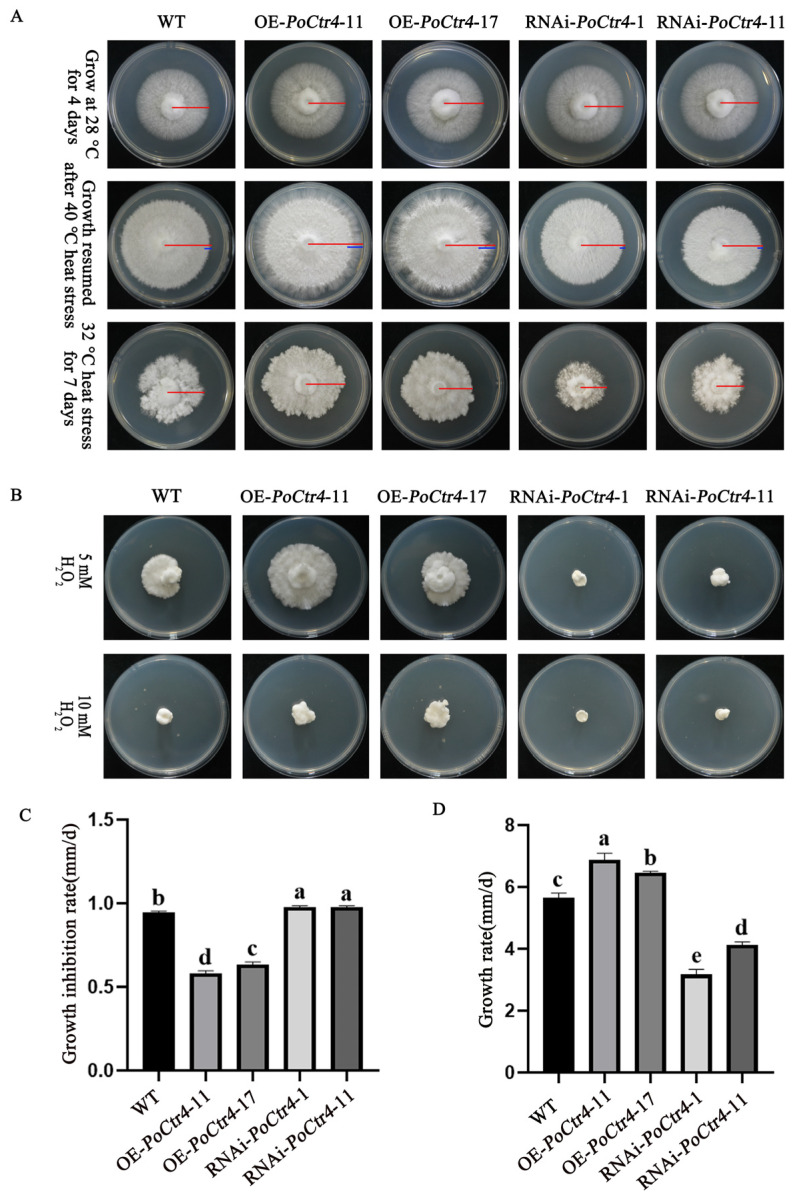
The OE of *PoCtr4* increased the tolerance of the strain to heat stress. (**A**) Comparison of the growth rates of the *PoCtr4* transformed strains after recovery from room temperature and heat stress and after heat stress at 32 °C. The red lines represent the radius of mycelial growth throughout the growth period, and blue lines represent the radius of mycelial growth during 48 h of recovery after heat stress. (**B**) The OE of *PoCtr4* enhances the tolerance of mycelia to H_2_O_2_. (**C**) Effects of the *PoCtr4*-transformed strains on the mycelial growth rate after heat stress. (**D**) Effects of the PoCtr4-transformed strains on the mycelial growth rate after heat stress at 32 °C. The values are the means ± SEs of three independent experiments. Different letters indicate significant differences for the comparison of samples (*p* < 0.05 according to Duncan’s test).

In this study, we tested the function of *PoCtr4* under heat stress at 32 °C. Compared with that of the WT strain, the colony diameter of the OE-*PoCtr4*-transformed strains grown at 32 °C for 7 d significantly increased, whereas the colony diameter of the RNAi-*PoCtr4* strain significantly decreased (Figure 10A) and the growth rates of the OE-*PoCtr4*-11 and OE-*PoCtr4*-17 strains increased by 17.50% and 14.00%, respectively, compared with those of the WT strain (Figure 10D). The growth rates of the RNAi-*PoCtr4*-1 and RNAi-*PoCtr4*-11 strains were 42.10% and 28.10% lower than those of the WT strains, respectively. OE-*PoCtr4* may play a positive regulatory role under heat stress at 32 °C and may exhibit a greater heat tolerance than the WT strain. RNAi-*PoCtr4* may play a negative regulatory role under heat stress at 32 °C. In the 40 °C recovery growth experiment, compared with that of the WT strain, the mycelial recovery growth rate of the OE-*PoCtr4* strain was significantly accelerated, and the growth inhibition rates of the OE-*PoCtr4*-11 and OE-*PoCtr4*-17 strains were reduced to 59.71% and 61.99%, respectively, compared with those of the WT strain (91.95%). In contrast, the recovery rates of the RNAi-*PoCtr4*-1 and RNAi-*PoCtr4*-11 strains did not significantly differ from those of the WT strains (Figure 10C). These findings suggest that the overexpression of *PoCtr4* positively regulates the mycelial response to heat stress at 40 °C. Further investigations were conducted to determine whether *PoCtr4* can increase the tolerance of mycelia to exogenous H_2_O_2_. Previous studies have shown that Cu can be transported by the Cu partner of superoxide dismutase (SOD) to the cytosolic enzyme SOD1, which can detoxify free radicals [31]. The results showed that, when the transformed strains were grown on a plate with a concentration of 5 mM H_2_O_2_ for 10 d, the OE strains presented an obvious tolerance to H_2_O_2_ compared with the WT strain, and the RNAi strains were significantly inhibited. When the concentration reached 10 mM, the RNAi strains and the WT strain did not significantly change, and the OE strains still grew (Figure 10B).

**Figure 11:**
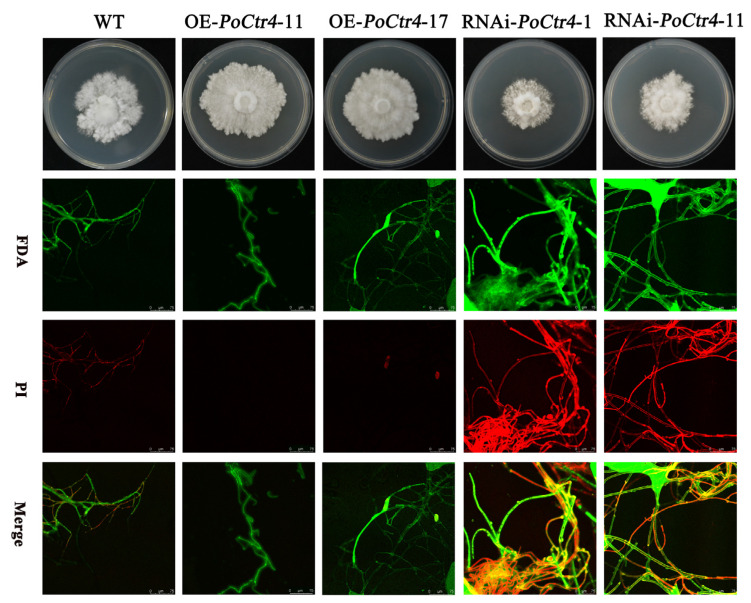
Effect of 32 °C stress on the integrity of the cell membrane of transformed strains on *P. ostreatus* mycelia in PDA plates. (fluorescein diacetate: FDA; propidium iodide: PI; potato dextrose agar: PDA).

Under 32 °C stress, the macroscopic characteristics of the agar plate indicated that the growth rate and colony density of the OE-transformed strains were significantly greater than those of the WT and RNAi strains. At the microscopic level, we investigated whether the transformed strains had an impact on cell membrane integrity under temperature stress. We used two fluorescent probes to study the effects of 32 °C heat stress treatment on the mycelia of *P. ostreatus* WT and transformed strains. As shown in Figure 11, compared with the control and RNAi-transformed strains, the OE-transformed strains presented an increase in the mycelial growth rate, an increase in mycelial density, and a decrease in red fluorescence. These findings suggest that OE-transformed strains can improve the integrity of cell membranes and increase the survival rate of *P. ostreatus* mycelial cells.

## 3. Discussion

Cu is an essential trace metal for living organisms. However, the copper transporter (COPT/Ctr) gene family plays a key role in maintaining metal homeostasis in living organisms [32]. Cu^2+^ is reduced to Cu^+^ by reductase on the surface of the cell membrane and is absorbed by Ctr4, thereby increasing the local metal concentration and participating in biological activities [33]. At present, there are also some reports about the Ctr family in the field of fungi. For example, in *Saccharomyces cerevisiae*, *Ctr1* and *Ctr3* have been identified as high-affinity copper transporters that localize to the plasma membrane [34]. In yeast, the high-copper-affinity system is activated via the *Mac1* transcription factor, which activates the transcriptional expressions of *Ctr1* and *Fre1* under copper-deficient conditions [35]. However, there are still few reports on Ctr in edible mushrooms. To further investigate the function of *Ctr* genes in edible mushrooms, we conducted an in-depth analysis of the *Ctr* genes family in *P. ostreatus*.

High-temperature stress is a nonbiological stress. In this study, we found that high-temperature stress can damage the cell membrane of the mycelia of *P. ostreatus*. Previous studies have shown that high-temperature stress damages the integrity of fungal cell walls [36]. Our research results, along with those of previous studies, indicate that high temperatures can cause cell damage. Cu plays complex roles in various cells [37], regulating the mechanisms of action of various cytokines and growth factors. Cu^+^ can regulate the level and activity of matrix metalloproteinases, thereby improving the efficiency of wound repair. In this study, heat stress led to cell membrane damage, and the addition of trace amounts of Cu^2+^ slowed cell membrane damage. Previous research has shown that Cu^+^ can act as an enzyme cofactor in detoxifying enzymes and play a role as a cofactor in iron transport. More importantly, the reduced availability of this metal can impair the function of many proteins involved in vital metabolic pathways. Cu can activate SOD1, thereby protecting the lysine biosynthesis pathway from oxidative damage [38], and high hepatic Cu may enhance the biosynthesis of a circulating ferroxidase, which potentiates iron release from stores [39]. Therefore, it is speculated that Cu^2+^ may increase the heat resistance of *P. ostreatus* mycelia by participating in multiple pathways.

The intracellular Ctr system plays a key role in maintaining Cu homeostasis [40], but very few studies have investigated this topic in edible fungi. Therefore, this systematic study of the Chr distribution, gene structure, conserved motifs, amino acid sequence alignment, transmembrane domain analysis, tertiary structure prediction, physicochemical property analysis, promoter prediction, and interspecific collinearity of the four *PoCtr* genes from the perspective of bioinformatics was performed on the basis of the Ctr family of *P. ostreatus*. The subcellular localization of PoCtrs was determined to play an important role in the study of their function, and the prediction of the four PoCtrs of *P. ostreatus* showed them to be located on the plasma membrane, which was consistent with the subcellular localization prediction of Ctrs in *Kandelia obovata* [28]. The MxxxM and GxxxG motifs are mostly present in transmembrane structures. The MxxxM motif regulates transport and absorption within Cu membranes. In multichannel membrane proteins, the GxxxG motif is important for protein folding [41]. Interestingly, our PoCtrs also had special motifs, such as MxxxM and GxxxG, indicating that these PoCtrs may play an important role in transmembrane transport. Promoter sequences that include cis-regulatory motifs and elements were utilized to enhance the understanding of how the *PoCtr* genes react to different environmental conditions. The group included factors that reacted to light, MeJA, auxin, defense and stress, drought, zein metabolism, gibberellin, abscisic acid, salicylic acid, anoxic, meristem expression, anaerobic induction, salicylic acid, low temperature, endosperm expression, and seed specificity. Gene promoters and their cis-acting elements play important roles in the activation and repression of gene expression for transcriptional regulation [42].

The Ctr family plays an important role in the growth and development of plants and fungi [43]. In *Populus trichocarpa*, *PtCtrs* may be involved in the balance of Cu and other metal concentrations and can also be used to produce *P. trichocarpa* varieties suitable for essential micronutrient deficiencies and metal-contaminated land [29]. The study of *MsCtr* family genes in alfalfa can effectively improve the understanding of Cu deficiency in the grass livestock chain [32]. In *A. fumigatus*, the Ctr family may collaborate with the regulation of the transcription factor *Mac1* to function and adapt to different Cu environments [44]. It can be inferred that Ctr genes play an important role in maintaining the steady state of Cu. In *Neurospora crassa*, the Cu concentration required for the downregulation of *Ctr* genes varies with different developmental stages [45]. Our study detected the expression levels of *PoCtr* in different developmental stages (mycelia, primordia, fruiting body, and spores). The research found that, compared with fruiting bodies, the expression patterns of the four *PoCtrs* at the spore stage were similar, with significantly increased expression levels at this stage. *PoCtr1* had the lowest expression level in the primordia stage compared to the other three stages. The expression patterns of *PoCtr2* and *PoCtr4* in the mycelia, primordia, and fruiting body stages were similar, with an initial increase followed by a decrease. There was no significant change in the expression level of PoCtr3 during the mycelia, primordia, and spore stages, and its expression level was the lowest during the fruiting body stage (Figure S4). Our results are similar to those of previous studies, indicating that there are differences in the functions of different Ctrs. In addition, we found that the RNAi of *PoCtr4* increased the degree of damage to the fungal cell membrane under heat stress. In contrast, the OE of *PoCtr4* weakened the cell membrane damage caused by heat stress. Previous studies have shown that, in *A. fumigatus*, double-deletion mutants of *CtrA2* and *CtrC* are sensitive to H_2_O_2_ and reduce SOD activity, laccase activity, and the intracellular copper content [24]. Therefore, it is speculated that *PoCtr4* may participate in the heat stress response process by regulating the concentration of Cu^+^, affecting the activity of detoxifying enzymes.

## 4. Materials and Methods

### 4.1. Strains

The *P. ostreatus* CCMSSC00389 strain was provided by the China Center for Mushroom Spawn Standards and Control. The WT and transformed strains were cultured on PDA.

### 4.2. Determination of Cell Membrane Integrity via the FDA/PI Dual-Color Fluorescence Method

Mycelia were grown at 28 °C for 4 d and then incubated at 32 °C, 36 °C, or 40 °C for 2 d. Mycelial samples were taken via the insertion method, and the staining solution was prepared with 20 µL of FDA. Then, 60 µL of PI and 920 µL of sterile water were added, and the mixture was mixed thoroughly. A total of 20 µL of staining solution was dropped onto a glass slide, and the cover slip was carefully picked up with tweezers. The cover slip was inverted onto the staining solution dropped on the glass slide, which was gently and evenly pressed. The samples were incubated in the dark at room temperature for 5 min to ensure complete staining. The samples were rinsed with distilled water one to two times, and the smear was observed. The incorporation of FDA during the staining process guaranteed a final concentration of 100 µg/mL, whereas the inclusion of PI resulted in a final concentration of 60 µg/mL. The PI-DNA complex had excitation and emission wavelengths of 535 nm and 615 nm, respectively. The excitation wavelength and emission wavelength indicated by the FDA were 488 nm and 530 nm, respectively. Images were obtained via laser confocal microscopy, and the images were saved.

### 4.3. Identification and Characterization of PoCtr Genes in P. ostreatus

The sequence of the *Ctr* genes was acquired from the National Biotechnology Information Center website (https://www.ncbi.nlm.nih.gov/gene/851129) (accessed on 15 June 2024) of *S. cerevisiae*. and the gene ID was 851129. The sequence of these genes were subsequently subjected to BLAST against the CCMSSC00389 genome database to identify four *PoCtr* genes. Four *PoCtr* family genes *(PoCtr1*, *PoCtr2*, *PoCtr3*, and *PoCtr4*) were identified and confirmed via conserved domain prediction via the NCBI database (https://www.ncbi.nlm.nih.gov/Structure/cdd/wrpsb.cgi) (accessed on 15 June 2024). The four PoCtr protein sequences can be accessed via online platforms (http://www.bioinformatics.org/sms/index.html) (accessed on 15 June 2024). The nucleotide sequences were then utilized to create primers for the amplification of the *PoCtr* genes. GFF files of *PoCtr* genes family member were extracted from the genomic GFF file of *P. ostreatus*, which included Chrs distribution information of the *PoCtr* family. The *PoCtr* genes family structure were predicted via the Gene Structure Display Server 2.0 Gao Lab-Bioinformatics and Computational Genomics (pku.edu.cn) (accessed on 15 June 2024).

### 4.4. Conserved Motif Analysis and Amino Acid Alignment of PoCtr Family Members

Four genes belonging to the *Ctr* family were identified within the genome of *P. ostreatus*. The online tool MEME (https://meme-suite.org/meme/) (accessed on 15 June 2024) was used to identify additional conserved motifs or regions within the protein sequences of the four PoCtr proteins. The letters “DNA”, “RNA”, or “protein” were sorted; locality distributions with zero or one occurrence per sequence (Zoops) were shown. The theme search mode was set to the default classic mode, with eight motifs configured manually within the application. The related master files were downloaded, and TBtools was used to display the MEME results. A phylogenetic tree was developed via the adjacency method in MEGA7 software. DNAMAN 8.0 software was used for multisequence alignment. ProtParam (http://web.ExPASy.org/protparam/) (accessed on 15 June 2024) was utilized for the determination of the physicochemical properties of sequences.

### 4.5. Transmembrane Structure Analysis and 3D Protein Structure Prediction

The *PoCtr* genes family was analyzed across the membrane domain via SOSUI, and the three-dimensional structure of the *PoCtr* genes family was visualized via SWISS-MODEL [46]. The structural domains of the PoCtr proteins were examined via the online resource available at (https://www.ncbi.nlm.nih.gov/Structure/cdd/wrpsb.cgi) (accessed on 15 June 2024). Additionally, a website (https://swissmodel.ExPASy.org/interactive) (accessed on 15 June 2024) was used to predict the 3D structures of the PoCtr proteins.

### 4.6. Analysis of the Ctr Family Promoter Sequence in P. ostreatus

A total of 2500 upstream sequences (2500 bp) corresponding to four members of the *PoCtrs* family were gathered from the *P. ostreatus* genome assembly database. The identification of CREs within these sequences was conducted via PlantCARE (http://bioinformatics.psb.ugent.be/webtools/plantcare/html/) (accessed on 15 June 2024). After each CRE motif was identified, the TBtools software was used to visualize the most common CRE motifs within the promoter of the *PoCtr* genes.

### 4.7. Interspecific Collinearity Analysis

In NCBI (https://www.ncbi.nlm.nih.gov/) (accessed on 15 June 2024), the genome files and gff annotation files of *Coprinopsis cinerea* and *Lentinus edodes* were downloaded, and TBtools was used to visualize the relationships among *P. ostreatus*, *L. edodes*, and *C. cinerea*.

### 4.8. Construction of OE-PoCtr4 and RNAi-PoCtr4 Plasmids

The construction of the *PoCtr4* gene OE plasmid was as follows. The original OE plasmid was generated in the laboratory with the restriction enzymes *Spe*I and *Psp*OMI. The cDNA of *PoCtr4* was then acquired through PCR, followed by cloning and insertion into a vector to create an overexpression plasmid that included the *PoCtr4* gene. To generate the *PoCtr4* gene RNAi plasmid, the cloned *PoCtr4* sequence was first homologously recombined onto the plasmid via the original RNAi plasmid stored in the laboratory with the *Spe*I and *Bgl*II restriction enzymes. The obtained plasmid was subsequently digested by *Spe*I and *Psp*OMI, and the cloned *PoCtr4*-anti fragment was subsequently inserted to construct the interference vector. Finally, the vector was introduced into *A. tumefaciens* (GV3101) [30]. The primers utilized in this study are presented in Supplementary Table S1.

### 4.9. Acquisition of Transformed Strains

First, *P. ostreatus* mycelium was seeded onto PDA plates and incubated in the dark at 28 °C for 4 d. Then, the mycelium pellet was cut at the edge of the colony to a diameter of 1 cm. The mycelium pellet was subsequently placed in CYM and left to stand in the dark at 28 °C for 2 d. *A. tumefaciens* containing OE and RNAi plasmids was expanded and cultured, then *A. tumefaciens* was collected in sterile tubes (50 mL capacity) by centrifugation at 4500 rpm and 4 °C for 15 min, and the waste solution was discarded. Then, the *A. tumefaciens* cells were subsequently suspended in induction medium (IM) and incubated for 5 h. Following this incubation, the mycelial pellets were placed onto IM (containing *A. tumefaciens*) and cocultured at 28 °C for an additional 5 h without shaking. The mycelial pellets were then transferred to IM solid media at 28 °C for a period of 3 d, after which they were moved to CYM media supplemented with 90 µg/mL of hygromycin (hyg) and 50 µg/mL of cefotaxime (cef). The transformants were successfully obtained after a period of 20–30 d.

### 4.10. qPCR

qPCR was used to examine the expression pattern of the *PoCt*r family in response to various temperature treatments, and mycelial samples were collected at different temperatures and in different time periods for gene expression detection. The *β*-*tubulin* gene was used as an internal control, and qPCR was used to analyze the mRNA expression levels of the *PoCtr* genes. The procedure for qPCR amplification was conducted as follows: amplification was conducted at 95 °C for 3 min, followed by amplification at 95 °C for 3 s, then at 60 °C for 32 s, repeated for a total of 40 cycles, concluding with amplification at 72 °C for 30 s. The analysis of relative gene expression was conducted via the 2^−∆∆CT^ method [47].

### 4.11. Heat Stress Treatment

The activated strains of WT, OE-*PoCtr4*-11, OE-*PoCtr4*-17, RNAi-*PoCtr4*-1, and RNAi-*PoCtr4*-11 were inoculated on PDA plates and cultured in the dark at 32 °C for 7 d. The growth of mycelia was subsequently observed, the growth rate was recorded, and photos were taken. The other group was cultured in the dark at 28 °C for 4 d, and after 2 d of stress at 40 °C, it was returned to 28 °C to observe the recovery of growth and record the growth situation.

### 4.12. H_2_O_2_ Susceptibility Assay

The sensitivity of the WT, OE, and RNAi-*PoCtr4* strains to H_2_O_2_ was determined according to previous methods [48]. Five-millimeter pellets of the test strains were introduced onto PDA plates that contained varying concentrations of H_2_O_2_ (0 mM, 5 mM, or 10 mM). After 10 d of incubation at 28 °C, the diameters of the strains were measured and photographed.

### 4.13. Statistical Analysis

GraphPad Prism 9 was used for the statistical analysis in this research. The values presented are the means ± SEs. The different letters among the samples indicate significant differences (*p* < 0.05 as determined by Duncan’s test).

## 5. Conclusions

**Figure 12:**
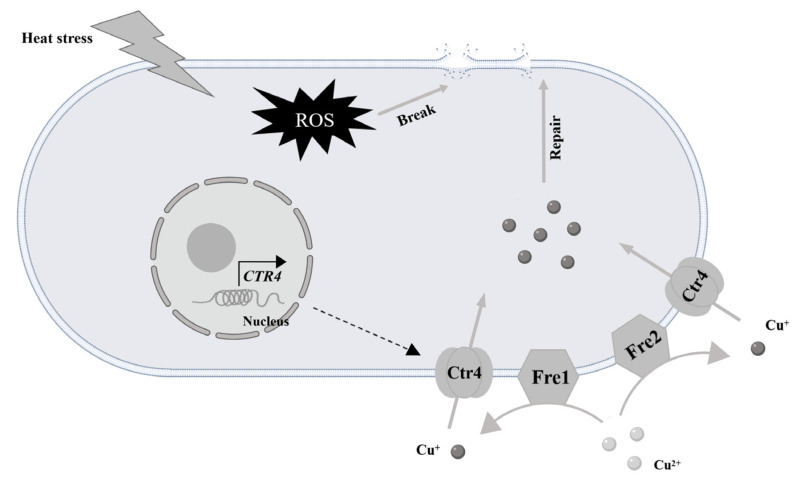
*PoCtr4* is involved in Cu^2+^ trafficking to repair cell membrane damage (The solid arrows represent the transcription process, while the dashed arrows represent the translation process).

In summary, high temperatures can damage the integrity of the cell membrane of *P. ostreatus*. Under mild temperature stress (32 °C), trace amounts of Cu can promote the growth of mycelia and increase their density. In addition, microscopic observation revealed that trace amounts of Cu contributed to the repair of the cell membrane damage caused by high temperatures. Furthermore, four *Ctr*-encoding genes were identified in the genome of *P. ostreatus*, and their functions in the growth and development of *P. ostreatus* were studied by constructing *PoCtr4*-transformed strains. The results showed that OE-*PoCtr4* enhanced mycelial tolerance to heat stress and H_2_O_2_. After high-temperature stress (40 °C), OE-*PoCtr4* promoted the recovery of mycelia. Under 32 °C stress, the OE of *PoCtr4* promoted mycelial growth, maintained cell membrane integrity, and reduced the degree of cell membrane damage caused by heat stress. It is speculated that OE-*PoCtr4* may maintain the integrity of the cell membrane and enhance the heat resistance of mycelia by regulating the homeostasis of Cu^+^ (Figure 12).

## Figures and Tables

**Table 1:** Detailed information on the *PoCtr* genes family identified in *P. ostreatus*.

Gene Name	Protein Length (aa)	Nucleotide Length (bp)	pI	MW	Number of Introns	Conserved Domain	Grand Average of Hydropathicity	Aliphatic Index	Localization
*PoCtr1*	174	521	9.60	19,056.33	2	Ctr	0.361	101.67	Plasma membrane
*PoCtr2*	207	620	8.48	22,530.53	2	Ctr	0.360	99.95	Plasma membrane
*PoCtr3*	194	581	8.88	21,000.53	3	Ctr	0.392	98.61	Plasma membrane
*PoCtr4*	678	2033	6.21	73,953.96	11	Ctr	0.086	91.03	Plasma membrane

## Data Availability

All the data generated or analyzed during this study are included in this published article and the Supplementary Information Files.

## Supplementary Materials

The supporting information can be downloaded at: https://www.mdpi.com/article/10.3390/ijms252212154/s1.
