# Supplementary material for: Family Identification and Functional Study of Copper Transporter Genes in Pleurotus ostreatus
Source: Int J Mol Sci. 2024 Nov 12;25(22):12154. doi: 10.3390/ijms252212154 (PMC11594920; doi:10.3390/ijms252212154)
Supplement: Supplementary file 1 [file ijms-25-12154-s001.zip › Supplementary material.pdf]

International Journal of Molecular Sciences

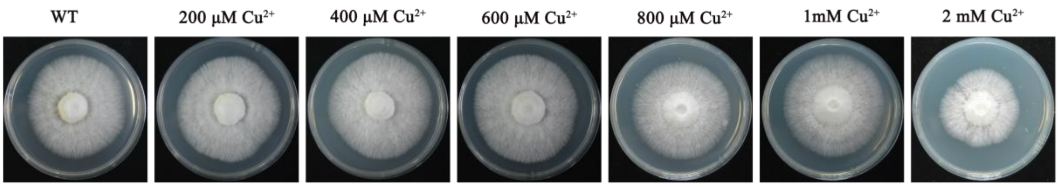

Figure S1. Effects of different concentrations of exogenous Cu^{2+} on the growth rate of mycelia

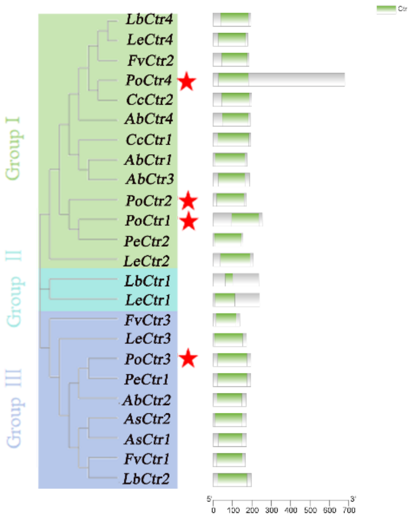

Figure S2. Prediction of conserved structural domains. The red stars represent the four *PoCtr* genes of *P. ostreatus* in this study.

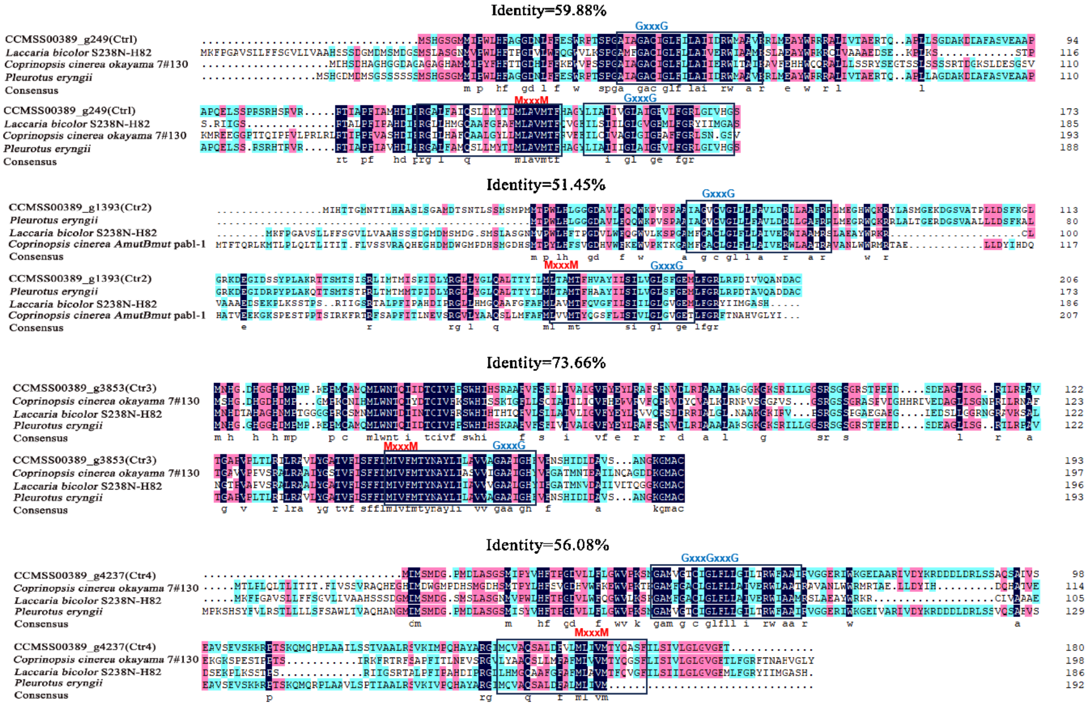

Figure S3. Amino acid sequences of Ctrs identified from different mushrooms. The red and blue text show the (MxxxM and GxxxG) motifs in the expected transmembrane region, respectively, and the MxxxM and GxxxG motifs are closely related to Cu transporter function in the transmembrane structural region. Black text represents 100%, pink text represents 75%, and light blue text represents 50% sequence similarity and sequence similarity. The black box represents the transmembrane structure region. Letters represent amino acid sequences.

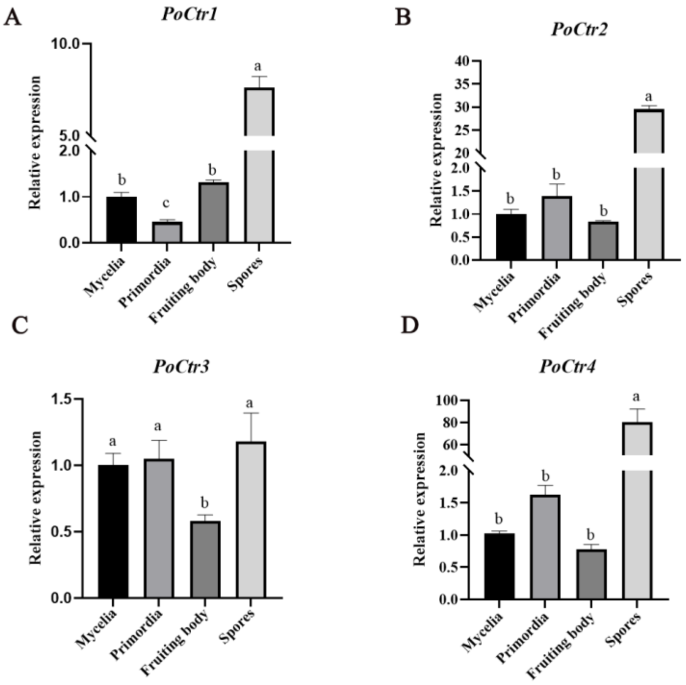

Figure S4. Expression levels of *PoCtr* at different stages of growth and development. (Different letters indicate significant differences in sample comparison, while the same letter indicates no significant differences in sample comparison (according to Duncan's test, $p<0.05$).

Table S1. Primer information relevant to this experimental study

Gene Name	Primer Name	Sequence (5′ to 3′)
β-tubulin	Forward	AGGCTTTCTTGCATTGGTACACGC
	Reverse	TATTCGCCTTCTTCCTCATCGGCA
hyg	Forward	CGACAGATCCGGTCGGCATCTACTCTATTTCTT
	Reverse	TCTCGTGCTTTCAGCTTCGATGTAGGAGGG
PoCtr1	Forward	ATGTCCCATGGCTCGGGCATGATG
	Reverse	CTAGTGAGAGCCATGGACATCACC
PoCtr2	Forward	ATGATCCACACGACGGGGATGAAC

	Reverse	TCAGCAACAAGCATCGTTAGCCTG
PoCtr3	Forward	ATGAACCACGGAGACCACGGCGGA
	Reverse	TCAATGGCACGCCATCCCCTTCCC
PoCtr4	Forward	GGCCAGCGGTTCCATGATTCCGTA
	Reverse	CCTGGGATAGAAAATCCGGGTAGA
OE-*PoCtr4*	Forward	GGTCAAAGTTACTAGGGCCAGCGGTTCCATGATTC
	Reverse	CAATTCTAGAGGGCCCCTGGGATAGAAAATCCGGGTAGAC
RNAi-*PoCtr4*-1	Forward	CCATCTCCTCAGATCGGCCAGCGGTTCCATGATTC
	Reverse	TAAGCTCTAAACTAGAAAGACAAGCGCTCATGATAACCAG
RNAi-*PoCtr4*-2	Forward	GCTTGTCTTTACTAGACACGAGGAACATGTAGACCATCC
	Reverse	CAATTCTAGAGGGCCGGCCAGCGGTTCCATGATTC
qPCR_*PoCtr1*	Forward	TTGATGGAAGCGTACTGGC
	Reverse	TGGTGAGGATAGTTCTTGAGGA
qPCR_*PoCtr2*	Forward	TGCTTGACCGTTTACTCGC
	Reverse	ATCCTTTTCTCCCATCGACG
qPCR_*PoCtr3*	Forward	GGTTTTCATGACATACAACGCG
	Reverse	TGGGAGTTGAAGACGAAATGG
qPCR_*PoCtr4*	Forward	GCGTTGGACTTTGTGCTTATG
	Reverse	ACCATATACCGACCAAATAGCG
